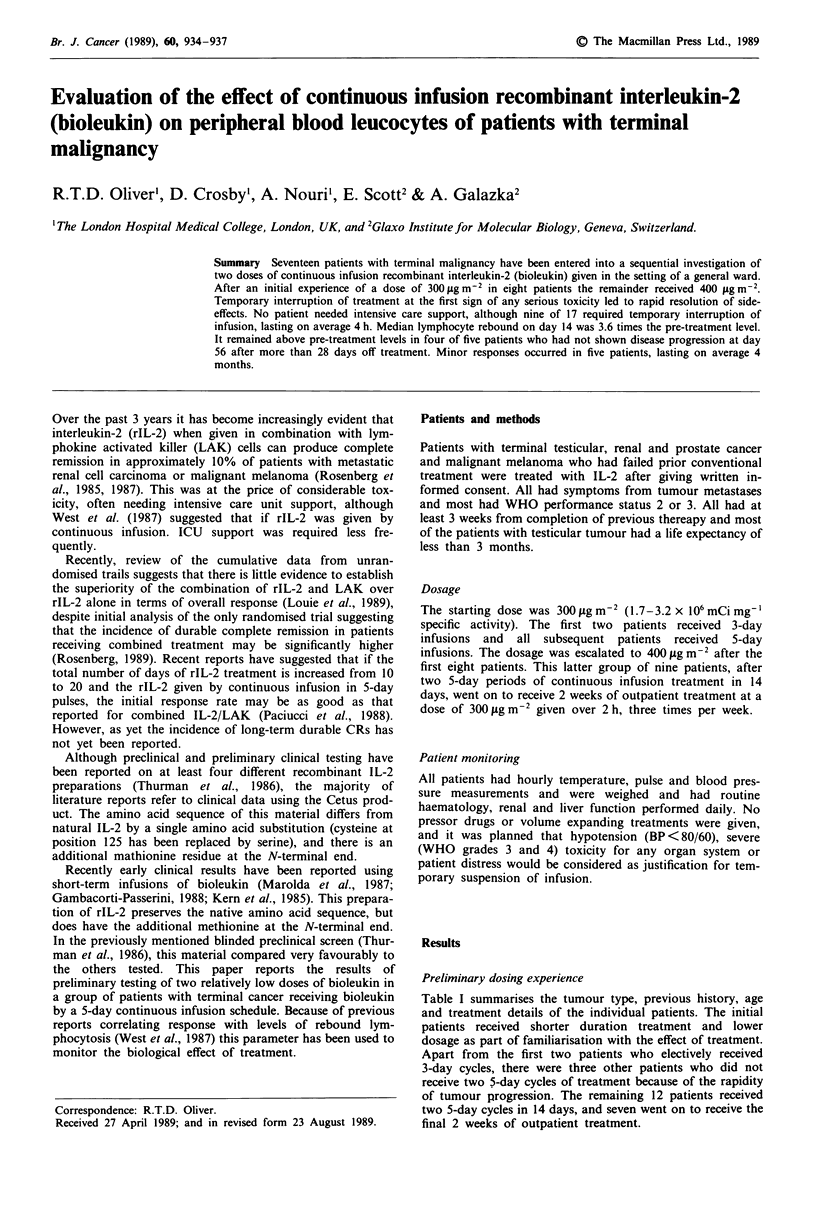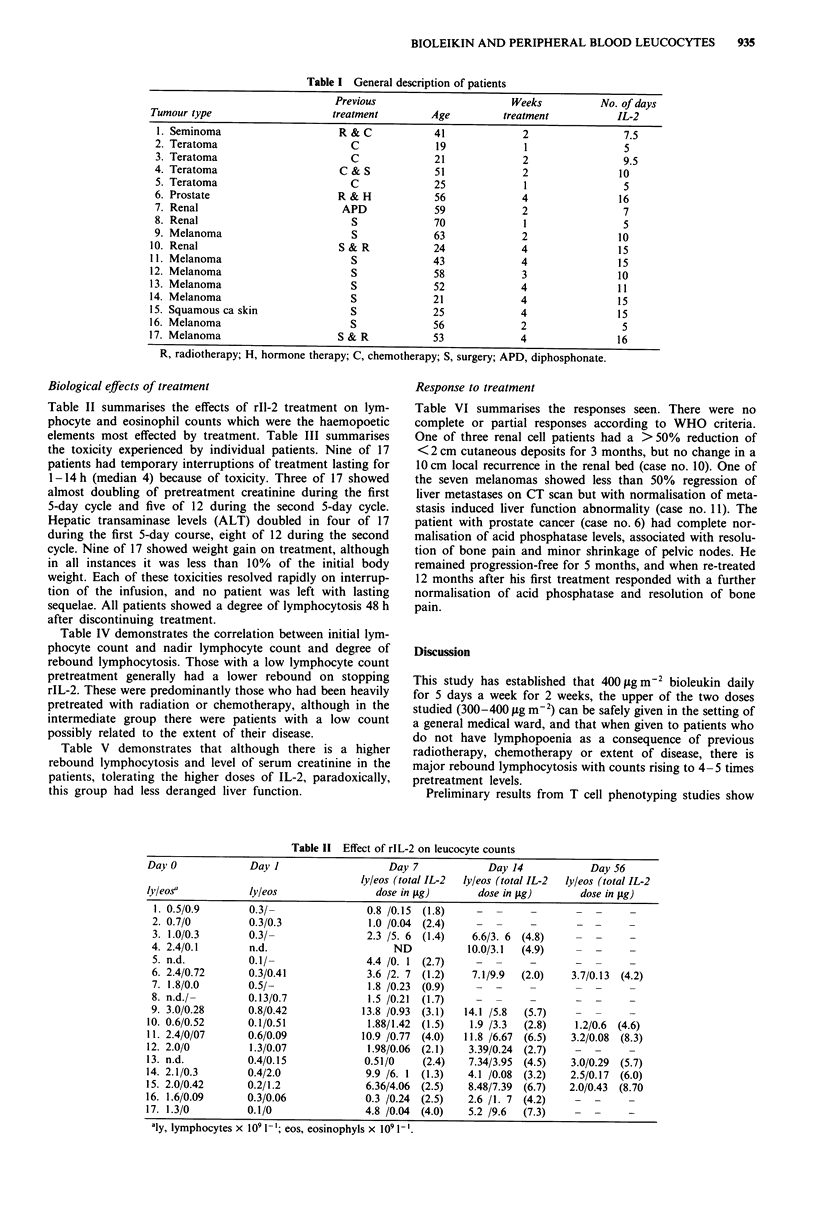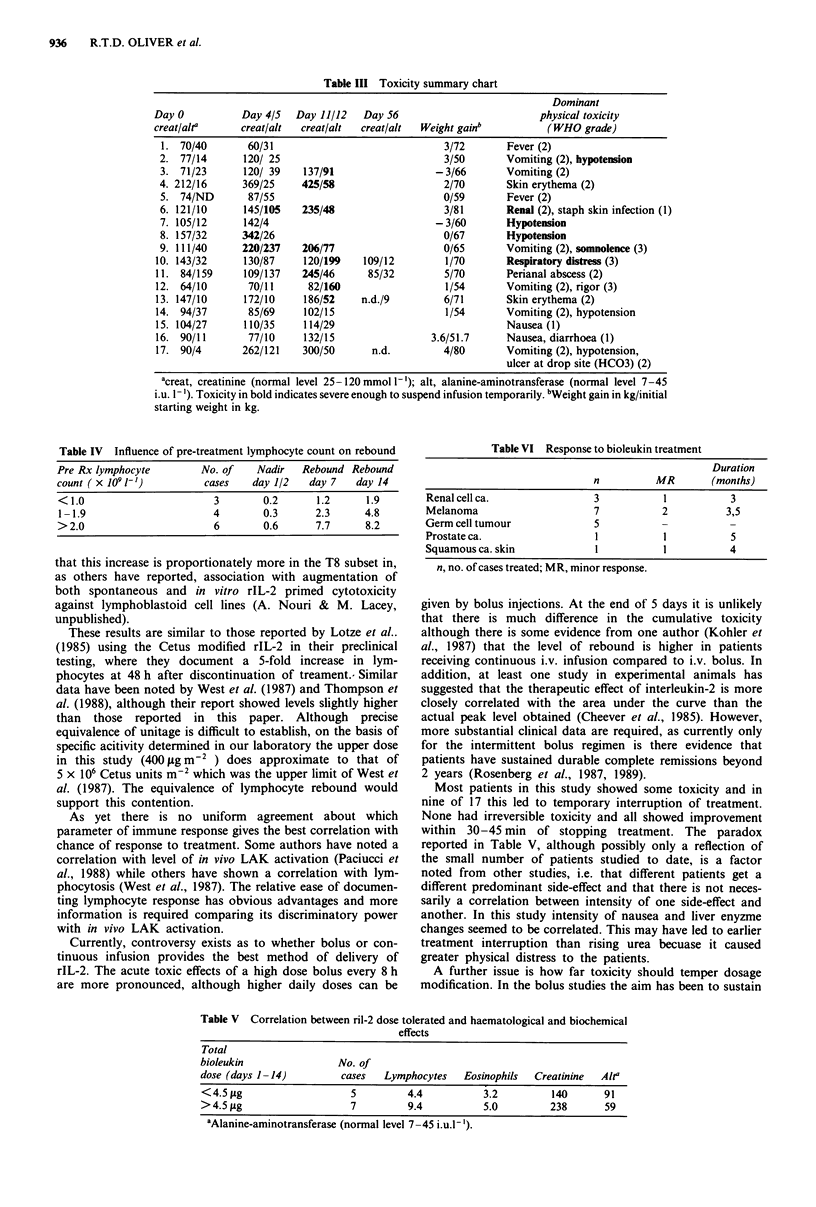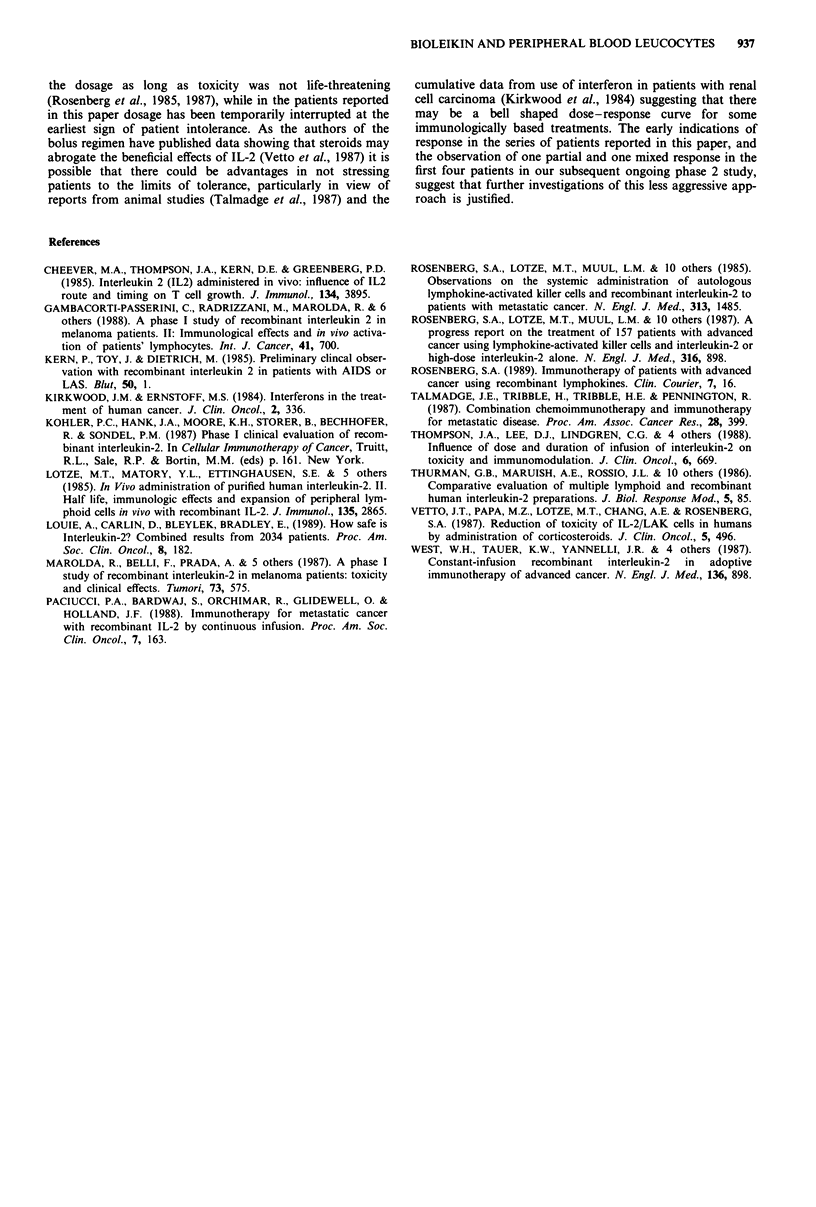# Evaluation of the effect of continuous infusion recombinant interleukin-2 (bioleukin) on peripheral blood leucocytes of patients with terminal malignancy.

**DOI:** 10.1038/bjc.1989.393

**Published:** 1989-12

**Authors:** R. T. Oliver, D. Crosby, A. Nouri, E. Scott, A. Galazka

**Affiliations:** London Hospital Medical College, UK.

## Abstract

Seventeen patients with terminal malignancy have been entered into a sequential investigation of two doses of continuous infusion recombinant interleukin-2 (bioleukin) given in the setting of a general ward. After an initial experience of a dose of 300 micrograms m-2 in eight patients the remainder received 400 micrograms m-2. Temporary interruption of treatment at the first sign of any serious toxicity led to rapid resolution of side-effects. No patient needed intensive care support, although nine of 17 required temporary interruption of infusion, lasting on average 4 h. Median lymphocyte rebound on day 14 was 3.6 times the pre-treatment level. It remained above pre-treatment levels in four of five patients who had no shown disease progression at day 56 after more than 28 days off treatment. Minor responses occurred in five patients, lasting on average 4 months.


Br. J. Cancer (1989), 60, 934–937 © The Macmillan Press Ltd., 1989

### Evaluation of the effect of continuous infusion recombinant interleukin-2 (bioleukin) on peripheral blood leucocytes of patients with terminal malignancy

R.T.D. Oliver[1], D. Crosby[1], A. Nouri[1], E. Scott[2] & A. Galazka[2]

[1]*The London Hospital Medical College, London, UK, and* [2]*Glaxo Institute for Molecular Biology, Geneva, Switzerland.*

**Summary** Seventeen patients with terminal malignancy have been entered into a sequential investigation of two doses of continuous infusion recombinant interleukin-2 (bioleukin) given in the setting of a general ward. After an initial experience of a dose of 300 $\mu g\ m^{-2}$ in eight patients the remainder received 400 $\mu g\ m^{-2}$. Temporary interruption of treatment at the first sign of any serious toxicity led to rapid resolution of side-effects. No patient needed intensive care support, although nine of 17 required temporary interruption of infusion, lasting on average 4 h. Median lymphocyte rebound on day 14 was 3.6 times the pre-treatment level. It remained above pre-treatment levels in four of five patients who had not shown disease progression at day 56 after more than 28 days off treatment. Minor responses occurred in five patients, lasting on average 4 months.

Over the past 3 years it has become increasingly evident that interleukin-2 (rIL-2) when given in combination with lymphokine activated killer (LAK) cells can produce complete remission in approximately 10% of patients with metastatic renal cell carcinoma or malignant melanoma (Rosenberg *et al.*, 1985, 1987). This was at the price of considerable toxicity, often needing intensive care unit support, although West *et al.* (1987) suggested that if rIL-2 was given by continuous infusion. ICU support was required less frequently.

Recently, review of the cumulative data from unrandomised trails suggests that there is little evidence to establish the superiority of the combination of rIL-2 and LAK over rIL-2 alone in terms of overall response (Louie *et al.*, 1989), despite initial analysis of the only randomised trial suggesting that the incidence of durable complete remission in patients receiving combined treatment may be significantly higher (Rosenberg, 1989). Recent reports have suggested that if the total number of days of rIL-2 treatment is increased from 10 to 20 and the rIL-2 given by continuous infusion in 5-day pulses, the initial response rate may be as good as that reported for combined IL-2/LAK (Paciucci *et al.*, 1988). However, as yet the incidence of long-term durable CRs has not yet been reported.

Although preclinical and preliminary clinical testing have been reported on at least four different recombinant IL-2 preparations (Thurman *et al.*, 1986), the majority of literature reports refer to clinical data using the Cetus product. The amino acid sequence of this material differs from natural IL-2 by a single amino acid substitution (cysteine at position 125 has been replaced by serine), and there is an additional mathionine residue at the *N*-terminal end.

Recently early clinical results have been reported using short-term infusions of bioleukin (Marolda *et al.*, 1987; Gambacorti-Passerini, 1988; Kern *et al.*, 1985). This preparation of rIL-2 preserves the native amino acid sequence, but does have the additional methionine at the *N*-terminal end. In the previously mentioned blinded preclinical screen (Thurman *et al.*, 1986), this material compared very favourably to the others tested. This paper reports the results of preliminary testing of two relatively low doses of bioleukin in a group of patients with terminal cancer receiving bioleukin by a 5-day continuous infusion schedule. Because of previous reports correlating response with levels of rebound lymphocytosis (West *et al.*, 1987) this parameter has been used to monitor the biological effect of treatment.

Correspondence: R.T.D. Oliver.
Received 27 April 1989; and in revised form 23 August 1989.

### Patients and methods

Patients with terminal testicular, renal and prostate cancer and malignant melanoma who had failed prior conventional treatment were treated with IL-2 after giving written informed consent. All had symptoms from tumour metastases and most had WHO performance status 2 or 3. All had at least 3 weeks from completion of previous thereapy and most of the patients with testicular tumour had a life expectancy of less than 3 months.

### *Dosage*

The starting dose was 300 $\mu g\ m^{-2}$ (1.7–3.2 × $10^6$ mCi $mg^{-1}$ specific activity). The first two patients received 3-day infusions and all subsequent patients received 5-day infusions. The dosage was escalated to 400 $\mu g\ m^{-2}$ after the first eight patients. This latter group of nine patients, after two 5-day periods of continuous infusion treatment in 14 days, went on to receive 2 weeks of outpatient treatment at a dose of 300 $\mu g\ m^{-2}$ given over 2 h, three times per week.

### *Patient monitoring*

All patients had hourly temperature, pulse and blood pressure measurements and were weighed and had routine haematology, renal and liver function performed daily. No pressor drugs or volume expanding treatments were given, and it was planned that hypotension (BP < 80/60), severe (WHO grades 3 and 4) toxicity for any organ system or patient distress would be considered as justification for temporary suspension of infusion.

### Results

### *Preliminary dosing experience*

Table I summarises the tumour type, previous history, age and treatment details of the individual patients. The initial patients received shorter duration treatment and lower dosage as part of familiarisation with the effect of treatment. Apart from the first two patients who electively received 3-day cycles, there were three other patients who did not receive two 5-day cycles of treatment because of the rapidity of tumour progression. The remaining 12 patients received two 5-day cycles in 14 days, and seven went on to receive the final 2 weeks of outpatient treatment.

**Table I** General description of patients

| Tumour type | Previous treatment | Age | Weeks treatment | No. of days IL-2 |
|---|---|---|---|---|
| 1. Seminoma | R & C | 41 | 2 | 7.5 |
| 2. Teratoma | C | 19 | 1 | 5 |
| 3. Teratoma | C | 21 | 2 | 9.5 |
| 4. Teratoma | C & S | 51 | 2 | 10 |
| 5. Teratoma | C | 25 | 1 | 5 |
| 6. Prostate | R & H | 56 | 4 | 16 |
| 7. Renal | APD | 59 | 2 | 7 |
| 8. Renal | S | 70 | 1 | 5 |
| 9. Melanoma | S | 63 | 2 | 10 |
| 10. Renal | S & R | 24 | 4 | 15 |
| 11. Melanoma | S | 43 | 4 | 15 |
| 12. Melanoma | S | 58 | 3 | 10 |
| 13. Melanoma | S | 52 | 4 | 11 |
| 14. Melanoma | S | 21 | 4 | 15 |
| 15. Squamous ca skin | S | 25 | 4 | 15 |
| 16. Melanoma | S | 56 | 2 | 5 |
| 17. Melanoma | S & R | 53 | 4 | 16 |

R, radiotherapy; H, hormone therapy; C, chemotherapy; S, surgery; APD, diphosphonate.

*Biological effects of treatment*

Table II summarises the effects of rIl-2 treatment on lymphocyte and eosinophil counts which were the haemopoetic elements most effected by treatment. Table III summarises the toxicity experienced by individual patients. Nine of 17 patients had temporary interruptions of treatment lasting for 1–14 h (median 4) because of toxicity. Three of 17 showed almost doubling of pretreatment creatinine during the first 5-day cycle and five of 12 during the second 5-day cycle. Hepatic transaminase levels (ALT) doubled in four of 17 during the first 5-day course, eight of 12 during the second cycle. Nine of 17 showed weight gain on treatment, although in all instances it was less than 10% of the initial body weight. Each of these toxicities resolved rapidly on interruption of the infusion, and no patient was left with lasting sequelae. All patients showed a degree of lymphocytosis 48 h after discontinuing treatment.

Table IV demonstrates the correlation between initial lymphocyte count and nadir lymphocyte count and degree of rebound lymphocytosis. Those with a low lymphocyte count pretreatment generally had a lower rebound on stopping rIL-2. These were predominantly those who had been heavily pretreated with radiation or chemotherapy, although in the intermediate group there were patients with a low count possibly related to the extent of their disease.

Table V demonstrates that although there is a higher rebound lymphocytosis and level of serum creatinine in the patients, tolerating the higher doses of IL-2, paradoxically, this group had less deranged liver function.

*Response to treatment*

Table VI summarises the responses seen. There were no complete or partial responses according to WHO criteria. One of three renal cell patients had a >50% reduction of <2 cm cutaneous deposits for 3 months, but no change in a 10 cm local recurrence in the renal bed (case no. 10). One of the seven melanomas showed less than 50% regression of liver metastases on CT scan but with normalisation of metastasis induced liver function abnormality (case no. 11). The patient with prostate cancer (case no. 6) had complete normalisation of acid phosphatase levels, associated with resolution of bone pain and minor shrinkage of pelvic nodes. He remained progression-free for 5 months, and when re-treated 12 months after his first treatment responded with a further normalisation of acid phosphatase and resolution of bone pain.

### Discussion

This study has established that 400 $\mu g\ m^{-2}$ bioleukin daily for 5 days a week for 2 weeks, the upper of the two doses studied (300–400 $\mu g\ m^{-2}$) can be safely given in the setting of a general medical ward, and that when given to patients who do not have lymphopoenia as a consequence of previous radiotherapy, chemotherapy or extent of disease, there is major rebound lymphocytosis with counts rising to 4–5 times pretreatment levels.

Preliminary results from T cell phenotyping studies show

**Table II** Effect of rIL-2 on leucocyte counts

| Day 0 ly/eos[a] | Day 1 ly/eos | Day 7 ly/eos (total IL-2 dose in µg) | Day 14 ly/eos (total IL-2 dose in µg) | Day 56 ly/eos (total IL-2 dose in µg) |
|---|---|---|---|---|
| 1. 0.5/0.9 | 0.3/– | 0.8 /0.15 (1.8) | – – – | – – – |
| 2. 0.7/0 | 0.3/0.3 | 1.0 /0.04 (2.4) | – – – | – – – |
| 3. 1.0/0.3 | 0.3/– | 2.3 /5. 6 (1.4) | 6.6/3. 6 (4.8) | – – – |
| 4. 2.4/0.1 | n.d. | ND | 10.0/3.1 (4.9) | – – – |
| 5. n.d. | 0.1/– | 4.4 /0. 1 (2.7) | – – – | – – – |
| 6. 2.4/0.72 | 0.3/0.41 | 3.6 /2. 7 (1.2) | 7.1/9.9 (2.0) | 3.7/0.13 (4.2) |
| 7. 1.8/0.0 | 0.5/– | 1.8 /0.23 (0.9) | – – – | – – – |
| 8. n.d./– | 0.13/0.7 | 1.5 /0.21 (1.7) | – – – | – – – |
| 9. 3.0/0.28 | 0.8/0.42 | 13.8 /0.93 (3.1) | 14.1 /5.8 (5.7) | – – – |
| 10. 0.6/0.52 | 0.1/0.51 | 1.88/1.42 (1.5) | 1.9 /3.3 (2.8) | 1.2/0.6 (4.6) |
| 11. 2.4/0/07 | 0.6/0.09 | 10.9 /0.77 (4.0) | 11.8 /6.67 (6.5) | 3.2/0.08 (8.3) |
| 12. 2.0/0 | 1.3/0.07 | 1.98/0.06 (2.1) | 3.39/0.24 (2.7) | – – – |
| 13. n.d. | 0.4/0.15 | 0.51/0 (2.4) | 7.34/3.95 (4.5) | 3.0/0.29 (5.7) |
| 14. 2.1/0.3 | 0.4/2.0 | 9.9 /6. 1 (1.3) | 4.1 /0.08 (3.2) | 2.5/0.17 (6.0) |
| 15. 2.0/0.42 | 0.2/1.2 | 6.36/4.06 (2.5) | 8.48/7.39 (6.7) | 2.0/0.43 (8.70 |
| 16. 1.6/0.09 | 0.3/0.06 | 0.3 /0.24 (2.5) | 2.6 /1. 7 (4.2) | – – – |
| 17. 1.3/0 | 0.1/0 | 4.8 /0.04 (4.0) | 5.2 /9.6 (7.3) | – – – |

[a]ly, lymphocytes × $10^9\ l^{-1}$; eos, eosinophyls × $10^9\ l^{-1}$.

Table III Toxicity summary chart

| Day 0 creat/alt[a] | Day 4/5 creat/alt | Day 11/12 creat/alt | Day 56 creat/alt | Weight gain[b] | Dominant physical toxicity (WHO grade) |
|---|---|---|---|---|---|
| 1. 70/40 | 60/31 | | | 3/72 | Fever (2) |
| 2. 77/14 | 120/ 25 | | | 3/50 | Vomiting (2), **hypotension** |
| 3. 71/23 | 120/ 39 | 137/**91** | | −3/66 | Vomiting (2) |
| 4. 212/16 | 369/25 | **425/58** | | 2/70 | Skin erythema (2) |
| 5. 74/ND | 87/55 | | | 0/59 | Fever (2) |
| 6. 121/10 | 145/**105** | **235/48** | | 3/81 | **Renal** (2), staph skin infection (1) |
| 7. 105/12 | 142/4 | | | −3/60 | **Hypotension** |
| 8. 157/32 | **342**/26 | | | 0/67 | **Hypotension** |
| 9. 111/40 | **220/237** | **206/77** | | 0/65 | Vomiting (2), **somnolence** (3) |
| 10. 143/32 | 130/87 | 120/**199** | 109/12 | 1/70 | **Respiratory distress** (3) |
| 11. 84/159 | 109/137 | **245**/46 | 85/32 | 5/70 | Perianal abscess (2) |
| 12. 64/10 | 70/11 | 82/**160** | | 1/54 | Vomiting (2), rigor (3) |
| 13. 147/10 | 172/10 | 186/**52** | n.d./9 | 6/71 | Skin erythema (2) |
| 14. 94/37 | 85/69 | 102/15 | | 1/54 | Vomiting (2), hypotension |
| 15. 104/27 | 110/35 | 114/29 | | | Nausea (1) |
| 16. 90/11 | 77/10 | 132/15 | | 3.6/51.7 | Nausea, diarrhoea (1) |
| 17. 90/4 | 262/121 | 300/50 | n.d. | 4/80 | Vomiting (2), hypotension, ulcer at drop site (HCO3) (2) |

[a]creat, creatinine (normal level 25–120 mmol $l^{-1}$); alt, alanine-aminotransferase (normal level 7–45 i.u. $l^{-1}$). Toxicity in bold indicates severe enough to suspend infusion temporarily. [b]Weight gain in kg/initial starting weight in kg.

Table IV Influence of pre-treatment lymphocyte count on rebound

| Pre Rx lymphocyte count ( $\times 10^9 l^{-1}$) | No. of cases | Nadir day 1/2 | Rebound day 7 | Rebound day 14 |
|---|---|---|---|---|
| <1.0 | 3 | 0.2 | 1.2 | 1.9 |
| 1–1.9 | 4 | 0.3 | 2.3 | 4.8 |
| >2.0 | 6 | 0.6 | 7.7 | 8.2 |

that this increase is proportionately more in the T8 subset in, as others have reported, association with augmentation of both spontaneous and *in vitro* rIL-2 primed cytotoxicity against lymphoblastoid cell lines (A. Nouri & M. Lacey, unpublished).

These results are similar to those reported by Lotze *et al.*. (1985) using the Cetus modified rIL-2 in their preclinical testing, where they document a 5-fold increase in lymphocytes at 48 h after discontinuation of treament.· Similar data have been noted by West *et al.* (1987) and Thompson *et al.* (1988), although their report showed levels slightly higher than those reported in this paper. Although precise equivalence of unitage is difficult to establish, on the basis of specific acitivity determined in our laboratory the upper dose in this study (400 $\mu g\,m^{-2}$ ) does approximate to that of $5 \times 10^6$ Cetus units $m^{-2}$ which was the upper limit of West *et al.* (1987). The equivalence of lymphocyte rebound would support this contention.

As yet there is no uniform agreement about which parameter of immune response gives the best correlation with chance of response to treatment. Some authors have noted a correlation with level of *in vivo* LAK activation (Paciucci *et al.*, 1988) while others have shown a correlation with lymphocytosis (West *et al.*, 1987). The relative ease of documenting lymphocyte response has obvious advantages and more information is required comparing its discriminatory power with *in vivo* LAK activation.

Currently, controversy exists as to whether bolus or continuous infusion provides the best method of delivery of rIL-2. The acute toxic effects of a high dose bolus every 8 h are more pronounced, although higher daily doses can be given by bolus injections. At the end of 5 days it is unlikely that there is much difference in the cumulative toxicity although there is some evidence from one author (Kohler *et al.*, 1987) that the level of rebound is higher in patients receiving continuous i.v. infusion compared to i.v. bolus. In addition, at least one study in experimental animals has suggested that the therapeutic effect of interleukin-2 is more closely correlated with the area under the curve than the actual peak level obtained (Cheever *et al.*, 1985). However, more substantial clinical data are required, as currently only for the intermittent bolus regimen is there evidence that patients have sustained durable complete remissions beyond 2 years (Rosenberg *et al.*, 1987, 1989).

Table VI Response to bioleukin treatment

| | *n* | *MR* | *Duration (months)* |
|---|---|---|---|
| Renal cell ca. | 3 | 1 | 3 |
| Melanoma | 7 | 2 | 3,5 |
| Germ cell tumour | 5 | – | – |
| Prostate ca. | 1 | 1 | 5 |
| Squamous ca. skin | 1 | 1 | 4 |

*n*, no. of cases treated; MR, minor response.

Most patients in this study showed some toxicity and in nine of 17 this led to temporary interruption of treatment. None had irreversible toxicity and all showed improvement within 30–45 min of stopping treatment. The paradox reported in Table V, although possibly only a reflection of the small number of patients studied to date, is a factor noted from other studies, i.e. that different patients get a different predominant side-effect and that there is not necessarily a correlation between intensity of one side-effect and another. In this study intensity of nausea and liver enyzme changes seemed to be correlated. This may have led to earlier treatment interruption than rising urea becuase it caused greater physical distress to the patients.

A further issue is how far toxicity should temper dosage modification. In the bolus studies the aim has been to sustain

Table V Correlation between ril-2 dose tolerated and haematological and biochemical effects

| Total bioleukin dose (days 1–14) | No. of cases | Lymphocytes | Eosinophils | Creatinine | Alt[a] |
|---|---|---|---|---|---|
| <4.5 µg | 5 | 4.4 | 3.2 | 140 | 91 |
| >4.5 µg | 7 | 9.4 | 5.0 | 238 | 59 |

[a]Alanine-aminotransferase (normal level 7–45 i.u.$l^{-1}$).

the dosage as long as toxicity was not life-threatening (Rosenberg *et al.*, 1985, 1987), while in the patients reported in this paper dosage has been temporarily interrupted at the earliest sign of patient intolerance. As the authors of the bolus regimen have published data showing that steroids may abrogate the beneficial effects of IL-2 (Vetto *et al.*, 1987) it is possible that there could be advantages in not stressing patients to the limits of tolerance, particularly in view of reports from animal studies (Talmadge *et al.*, 1987) and the cumulative data from use of interferon in patients with renal cell carcinoma (Kirkwood *et al.*, 1984) suggesting that there may be a bell shaped dose–response curve for some immunologically based treatments. The early indications of response in the series of patients reported in this paper, and the observation of one partial and one mixed response in the first four patients in our subsequent ongoing phase 2 study, suggest that further investigations of this less aggressive approach is justified.

### References

CHEEVER, M.A., THOMPSON, J.A., KERN, D.E. & GREENBERG, P.D. (1985). Interleukin 2 (IL2) administered in vivo: influence of IL2 route and timing on T cell growth. *J. Immunol.*, **134**, 3895.

GAMBACORTI-PASSERINI, C., RADRIZZANI, M., MAROLDA, R. & 6 others (1988). A phase I study of recombinant interleukin 2 in melanoma patients. II: Immunological effects and *in vivo* activation of patients' lymphocytes. *Int. J. Cancer*, **41**, 700.

KERN, P., TOY, J. & DIETRICH, M. (1985). Preliminary clincal observation with recombinant interleukin 2 in patients with AIDS or LAS. *Blut*, **50**, 1.

KIRKWOOD, J.M. & ERNSTOFF, M.S. (1984). Interferons in the treatment of human cancer. *J. Clin. Oncol.*, **2**, 336.

KOHLER, P.C., HANK, J.A., MOORE, K.H., STORER, B., BECHHOFER, R. & SONDEL, P.M. (1987) Phase I clinical evaluation of recombinant interleukin-2. In *Cellular Immunotherapy of Cancer*, Truitt, R.L., Sale, R.P. & Bortin, M.M. (eds) p. 161. New York.

LOTZE, M.T., MATORY, Y.L., ETTINGHAUSEN, S.E. & 5 others (1985). *In Vivo* administration of purified human interleukin-2. II. Half life, immunologic effects and expansion of peripheral lymphoid cells *in vivo* with recombinant IL-2. *J. Immunol.*, **135**, 2865.

LOUIE, A., CARLIN, D., BLEYLEK, BRADLEY, E., (1989). How safe is Interleukin-2? Combined results from 2034 patients. *Proc. Am. Soc. Clin. Oncol.*, **8**, 182.

MAROLDA, R., BELLI, F., PRADA, A. & 5 others (1987). A phase I study of recombinant interleukin-2 in melanoma patients: toxicity and clinical effects. *Tumori*, **73**, 575.

PACIUCCI, P.A., BARDWAJ, S., ORCHIMAR, R., GLIDEWELL, O. & HOLLAND, J.F. (1988). Immunotherapy for metastatic cancer with recombinant IL-2 by continuous infusion. *Proc. Am. Soc. Clin. Oncol.*, **7**, 163.

ROSENBERG, S.A., LOTZE, M.T., MUUL, L.M. & 10 others (1985). Observations on the systemic administration of autologous lymphokine-activated killer cells and recombinant interleukin-2 to patients with metastatic cancer. *N. Engl. J. Med.*, **313**, 1485.

ROSENBERG, S.A., LOTZE, M.T., MUUL, L.M. & 10 others (1987). A progress report on the treatment of 157 patients with advanced cancer using lymphokine-activated killer cells and interleukin-2 or high-dose interleukin-2 alone. *N. Engl. J. Med.*, **316**, 898.

ROSENBERG, S.A. (1989). Immunotherapy of patients with advanced cancer using recombinant lymphokines. *Clin. Courier*, **7**, 16.

TALMADGE, J.E., TRIBBLE, H., TRIBBLE, H.E. & PENNINGTON, R. (1987). Combination chemoimmunotherapy and immunotherapy for metastatic disease. *Proc. Am. Assoc. Cancer Res.*, **28**, 399.

THOMPSON, J.A., LEE, D.J., LINDGREN, C.G. & 4 others (1988). Influence of dose and duration of infusion of interleukin-2 on toxicity and immunomodulation. *J. Clin. Oncol.*, **6**, 669.

THURMAN, G.B., MARUISH, A.E., ROSSIO, J.L. & 10 others (1986). Comparative evaluation of multiple lymphoid and recombinant human interleukin-2 preparations. *J. Biol. Response Mod.*, **5**, 85.

VETTO, J.T., PAPA, M.Z., LOTZE, M.T., CHANG, A.E. & ROSENBERG, S.A. (1987). Reduction of toxicity of IL-2/LAK cells in humans by administration of corticosteroids. *J. Clin. Oncol.*, **5**, 496.

WEST, W.H., TAUER, K.W., YANNELLI, J.R. & 4 others (1987). Constant-infusion recombinant interleukin-2 in adoptive immunotherapy of advanced cancer. *N. Engl. J. Med.*, **136**, 898.